# Novel I_1_-Imidazoline Agonist S43126 Augment Insulin Secretion in Min6 Cells

**DOI:** 10.4172/2155-6156.1000183

**Published:** 2012-04-25

**Authors:** Jerusalem Tesfai, Louis Crane, Genevieve Baziard-Mouysset, Lincoln P. Edwards

**Affiliations:** 1 Department of Biochemistry, Loma Linda University School of Medicine, Loma Linda, CA, USA; 2 Laboratoire de Chimie Pharmaceutique, Universite Paul Sabatier, Faculté de Pharmacie, USA; 3 Center for Dental Research, School of Dentistry and School of Medicine, Loma Linda University, Loma Linda, CA, USA

## Abstract

The I_1_-imidazoline receptor is a novel drug target for hypertension and insulin resistance which are major disorders associated with Type II diabetes. In the present study, we examined the effects of a novel imidazoline agonist S43126 on calcium fluxes and insulin secretion from Min6 β-cells. We also examined the effects of S43126 on the induction of IRAS, and phosphorylation of components in the I_1_-imidazoline signaling pathways, namely ERK and PKB. Min6 β-cells were treated with varying doses of S43126 [10^−8^M to 10^−5^M] for various time (5–60mins). S43126 at higher dose [10^−5^M] stimulated insulin secretion under elevated glucose concentration compared to basal. In addition, insulin secretion and Ca^2+^ influx mediated by S43126 [10^−5^M] were decreased following co-treatment with efaroxan (I_1_-antagonist) and nifedipine (L-type voltage-gated Ca^2+^-channel blocker) at various times (5–60mins). Furthermore, S43126 at [10^−5^M] increased Ca^2+^ oscillation, [Ca^2+^] and ^45^Ca^2+^ uptake in a time and dose-dependent manner. Moreover, Western blot analysis of treated samples showed that S43126 caused an increased protein expression of IRAS as well as phosphorylation of both ERK1/2 and PKB in a concentration-dependent manner. We conclude that S43126 exerts its insulinotropic effect in a glucose dependent manner by a mechanism involving L-type calcium channels and imidazoline I_1_-receptors.

## Introduction

Insulin resistance and hypertension are commonly associated with metabolic syndrome, which affects over 75 million Americans, and type 2 diabetes which affects over 18 million Americans [1]. Pharmacologic treatment of many type 2 diabetic patients requires separate agents for treating hyperglycemia, and hypertension. This results in patients having to take multiple medications, which negatively impact patient compliance and increases the risk for drug interaction. In response to this growing health care problem, compounds that have the ability to counter both hyperglycemia and hypertension would positively impact compliance and be an asset to patients.

Pharmacologic criteria have defined three main types of imidazoline receptors: the I_1_ subtype is labeled by [^3^H] clonidine and the I_2_ subtype is labeled by [^3^H] idazoxan [2,3]. A third pharmacologically distinct entity, the I_3_ subtype, is found in the pancreas and is involved in regulation of insulin secretion [4]. Functionally, I_2_-imidazoline sites seem to play a role in depression as the density of I_2_-sites were altered in suicide/depressive patients and the I_2_-selective compound 2-(2-benzofuranyl)-2-imidazoline (2-BFI) demonstrated antidepressant-like effects in mice according to the tail suspension test and the forced swim test [5]. The I_2_-site is also an emerging drug target for pain treatment [6] and I_2_-agonists have been shown to enhance the antinociceptive effects of opioids [7]. There is an emerging role for I_2_-agonists in the regulation of glucose homeostasis. Cerebral injections of agmatine reduced plasma glucose levels in streptozotocin-induced diabetic (STZ-diabetic) rats by a mechanism not involving insulin secretion but activation of I_2_-imidazoline receptors [8]. It was subsequently shown that peripheral administration of agmatine caused activation of I_2_-receptors in the adrenal medulla to enhance secretion of β-endorphins, leading to activation of μ-opioid receptors, and lower glucose levels [9]. Additionally it was shown that in rats where insulin resistance was induced by a high fructose diet, agmatine (1mg/kg) ameliorated the insulin resistance by a mechanism involving I_2_-imidazoline receptors [10].

Imidazoline compounds, which are agonists at the I_1_-imidazoline receptor (I_1_R) present in the rostral ventrolateral medulla (RVLM) region of brain [11,12] act centrally to lower blood pressure. Clinical and basic findings also indicate a role for I_1_-imidazoline agonists in the treatment of insulin resistance and diabetics with hypertension [13,14].

Several studies have shown that compounds containing the imidazoline moiety are potent stimulators of insulin secretion from pancreatic β-cells [15–19]. The mechanisms by which imidazoline compounds promote insulin secretion have not been fully elucidated. Classical imidazoline compounds mimic the actions of sulfonylurea drugs and interact directly with the pore-forming component (Kir6.2) of the ATP-sensitive potassium (K_ATP_) channel to promote channel closure, membrane depolarization, Ca^2+^ influx and insulin secretion [15,17,20,21]. These agents also have a direct effect on exocytosis. Other imidazoline compounds have been shown to have no effect on the K_ATP_ channel, but exert their insulinotropic effects only if glucose concentration is elevated [18]. Some agents show a dependence on protein kinase A and C to exert their insulinotropic effects [18]

We have previously shown that S43126 ( pK_i_ I_1_=7.46, pK_i_ I_2_=8.28, pK_i_ α_1_<5 and pK_i_α_2_<5) a novel imidazoline compound with close binding affinities for both I_1_ and I_2_ imidazoline binding sites [22], lowers blood pressure when injected into the RVLM of spontaneously hypertensive rats. This compound does not contract rat tail arterial strips suggesting that it is inactive at alpha adrenergic receptors [23]. In this study we describe the effects of S43126 on calcium fluxes, insulin secretion and glucose uptake. Imidazoline compounds may prove useful in treating diabetics with hypertension

## Materials and Methods

### Antibodies and reagents

Primary antibodies used were IRAS, β-actin, p44/42 MAP kinase, phospho-p44/42 MAP kinase (Thr-202/Tyr-204), Akt, phospho-Akt (Ser473) antibody diluted 1:1000, which were detected using a secondary antibody (HRP linked anti-rabbit IgG), diluted 1:2000 and enhanced chemiluminescence (ECL, Amersham Pharmacia Biotech). Treated cells were lysed and aliquots were subjected to western blotting using appropriate antibodies.

### Cell culture and drug treatment

Min6 β-cells were cultured in DMEM (Cellgro) supplemented with 15% FBS, 5ml penicillin/streptomycin solution (Sigma), 1 μL β-mercaptoethanol (Sigma) and maintained in the presence of 5% CO_2_ at 37°C. Min6 β-cells were treated with varying doses of S43126 [10^−5^ M–10^−8^M] for different times, in the presence or absence of I_1_-imidazoline receptor blocker efaroxan [100μM] or L-type calcium channel blocker nifedipine [10μM].

### Single-cell microfluorimetry

Min6 β-cells were seeded on 25 mm glass coverslips in 35 mm plastic dishes. Twenty-four hours before imaging, culture medium was replaced with serum free medium. On the day of the experiment, culture medium was removed; cells were washed twice with Krebs-Ringer bicarbonate (KRB) buffer, pH 7.5, and containing 2.8 mM glucose. Washed cells were incubated with KRB/glucose for 2 hours at 37°C, 5% CO_2._ In preparation for imaging, cells grown on the coverslip were loaded with 5 ul of Ca^2+^ Fluorophore, Fura2/AM for 45 mins at room temperature. The coverslips were rinsed in KRB buffers and placed in a 300 μl steel chamber attached to the stage of a Nikon inverted microscope (Nikon Instruments, Tokyo, Japan). A low pressure, rapid super fusion system (3 ml/min) was used to change solutions (i.e. S43126, glucose and KRB buffers) in the bath chamber. Ratios of 510 nm emission at 340 nm vs. 380 nm excitation wavelengths were acquired every second by photometric Cool Snap 12-bit digital camera. To facilitate comparison between experiments, data are expressed as 340/380-nm ratios normalized to the baseline ratio [24].

### ^45^Ca^2+^ influx

^45^Ca influx was determined by a modification of the methods of Edwards et al. and Brigand et al. [25,26]. Min6 β-cell in 6-well plates were treated as above but on the day of the experiment, cells were preincubated for 10 min at 37°C, 5% CO_2_ in KRB. The preincubation solution was then replaced with KRB containing 3 uCi/ml ^45^CaCl_2_, in the presence or absence of S43126 [10^−5^M], efaroxan [100 μM] or nifedipine [10 μM] for various times (0–30 mins). Treated cells were quickly washed five times with ice-cold saline and then solubilized in 1 ml KRB containing 0.1% Triton for 4 hours at room temperature. Aliquots (200 μl) of the solution were then assayed for ^45^Ca^2+^ content after the addition of 5 ml liquid scintillation medium (Perkin Elmer). Protein concentrations were determined using the BioRad DC Protein assay reagents (BioRad, Hercules, CA).

### Insulin secretion

Monolayers of Min6 β-cells were seeded 3 days before each series of studies in 24-well plates at a density of 500,000 cells per well. 12 hours before each experiment, the culture medium was replaced with serum free medium. On the day of the experiment, the cells were washed twice with KRB buffer, pH 7.5, containing 0.1% BSA (KRB-BSA). Cells were then preincubated for 2 hrs in KRB-BSA containing 2.8 mM glucose at 37°C, 5% CO_2_ followed by incubated for 2 hours in KRB-BSA containing various concentration of effectors (S43126, efaroxan and nifedipine) and glucose (2.8mM or 16.7mM) [13]. After incubation, the medium was collected, centrifuged at 600 X g for 10 mins and stored at −20°C. Insulin release was measured using Mercodia ultrasensitive mouse insulin enzyme-linked immunosorbent assay (ELISA) kit (Mercodia AB, Uppsala, Sweden). Protein concentrations were determined using the BioRad DC Protein assay reagents (BioRad, Hercules, CA).

### Statistical analysis

Data was analyzed using two-way analysis of variance (ANOVA) or Tukey’s test for pair-wise multiple comparisons to identify significant differences (P>0.05) between individual samples. Percent blockade was calculated using the formula % blockade = (S - [S+N])/(S - baseline) * 100 (Figure 3 and 4).

## Results

### Effects of S43126 on insulin secretion

**Figure 1:**
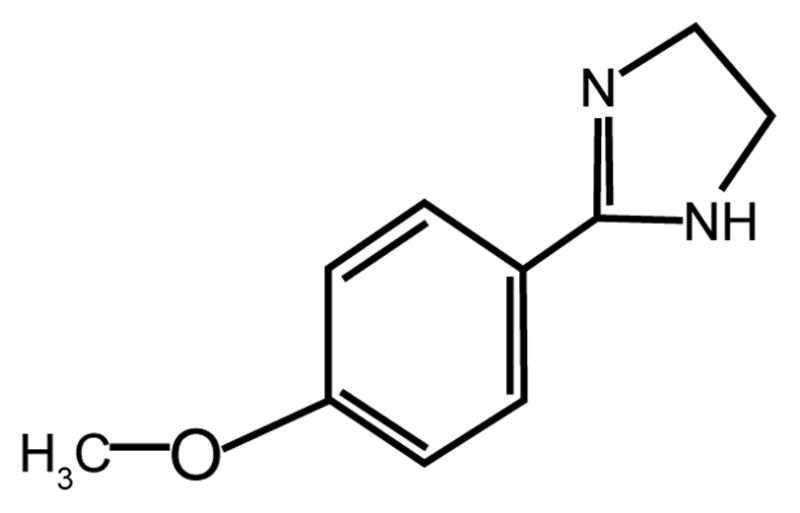
Chemical Structure of the Novel Imidazoline Compound, S43126.

**Figure 2:**
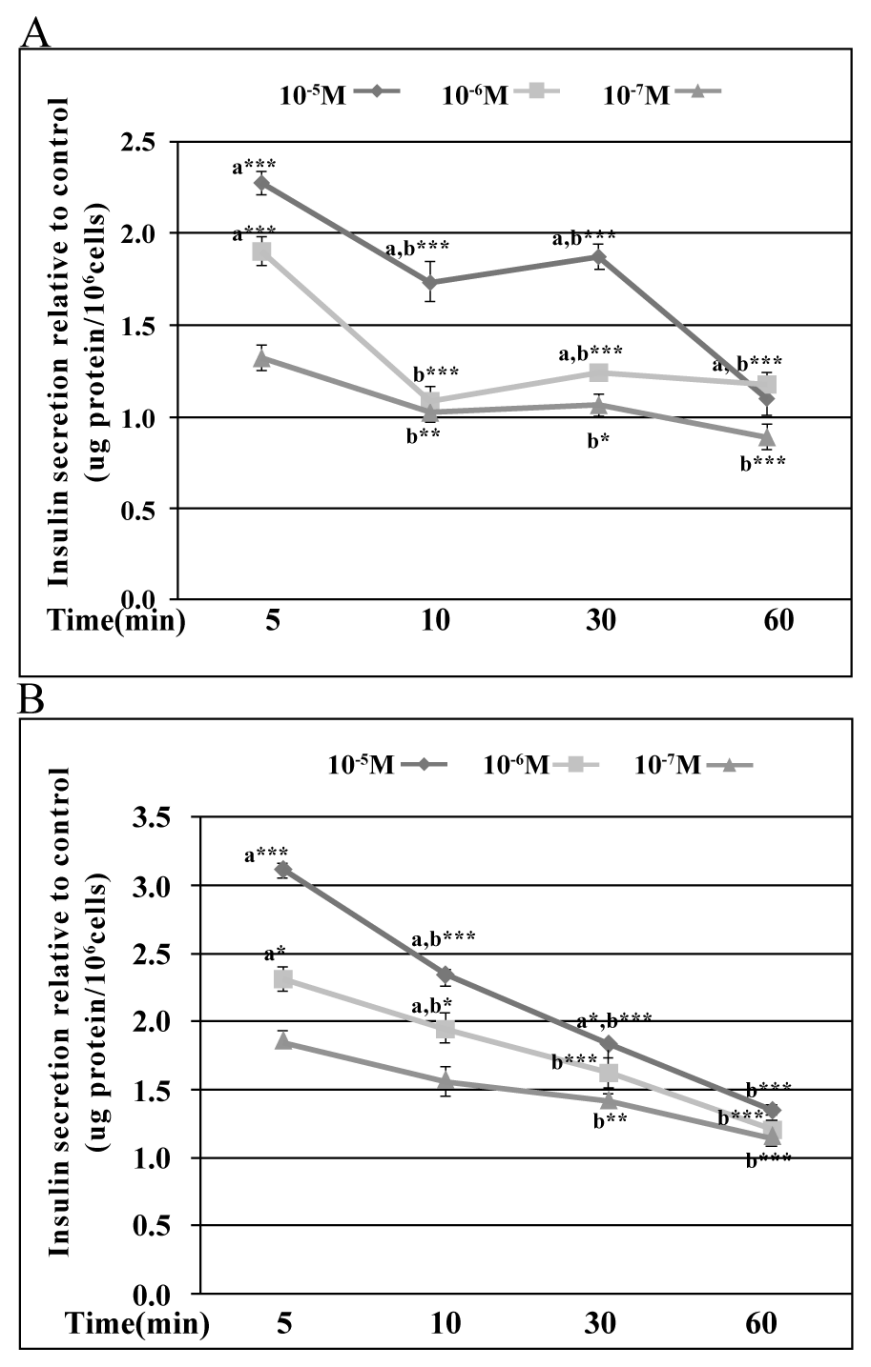
Stimulatory effects of S43126 on insulin release from Min6 cells. S43126 [10^−5^M] at 5mins induced the highest insulin release (2.3 fold) and 3.1 fold under conditions of (A) low glucose ( 2.8 mM) and (B) high glucose (16.8 mM) respectively, relative to control. (A) Data are presented as mean percent change +/− standard error from three separate experiments run in duplicate. (***a***= ***p< 0.001 same time point, compared to [10^−7^M] and ***b***= ***p< 0.001 same dose, different time compared to 5mins, ANOVA) (B) Data are presented as mean percent change +/− standard error from three separate experiments run in duplicate. (***a***= **p< 0.01, ***p< 0.001 same time point, compared to [10^−7^M] ***b***= **p< 0.01, ***p< 0.001 same dose, different time compared to 5 mins, two-way ANOVA).

We examined the ability of S43126 [10^−7^M–10^−5^M] to induce insulin secretion directly from Min6 β-cells at various times (5–60mins) under conditions of basal glucose (2.8 mM) and high glucose (16.7 mM). S43126, a novel I_1_-imidazoline agonist (Figure 1), induced a dose-dependent release of insulin at 5 mins, 10 mins and 30 mins under conditions of basal glucose (Figure 2A), and under high glucose (Figure 2B). At 5 mins and 10 mins, the amount of insulin release caused by a particular dose of S43126 was greater under conditions of high glucose than at basal glucose. Under conditions of basal glucose, the maximal release of insulin was 2.3 fold, seen at 5 mins of treatment with 10^−5^M S43126. Under conditions of high glucose, the maximal release of insulin was 3.1 fold, seen at 5 mins of treatment with 10^−5^M S43126. Relative insulin secretion decreased with time following stimulation under basal and high glucose.

**Figure 3:**
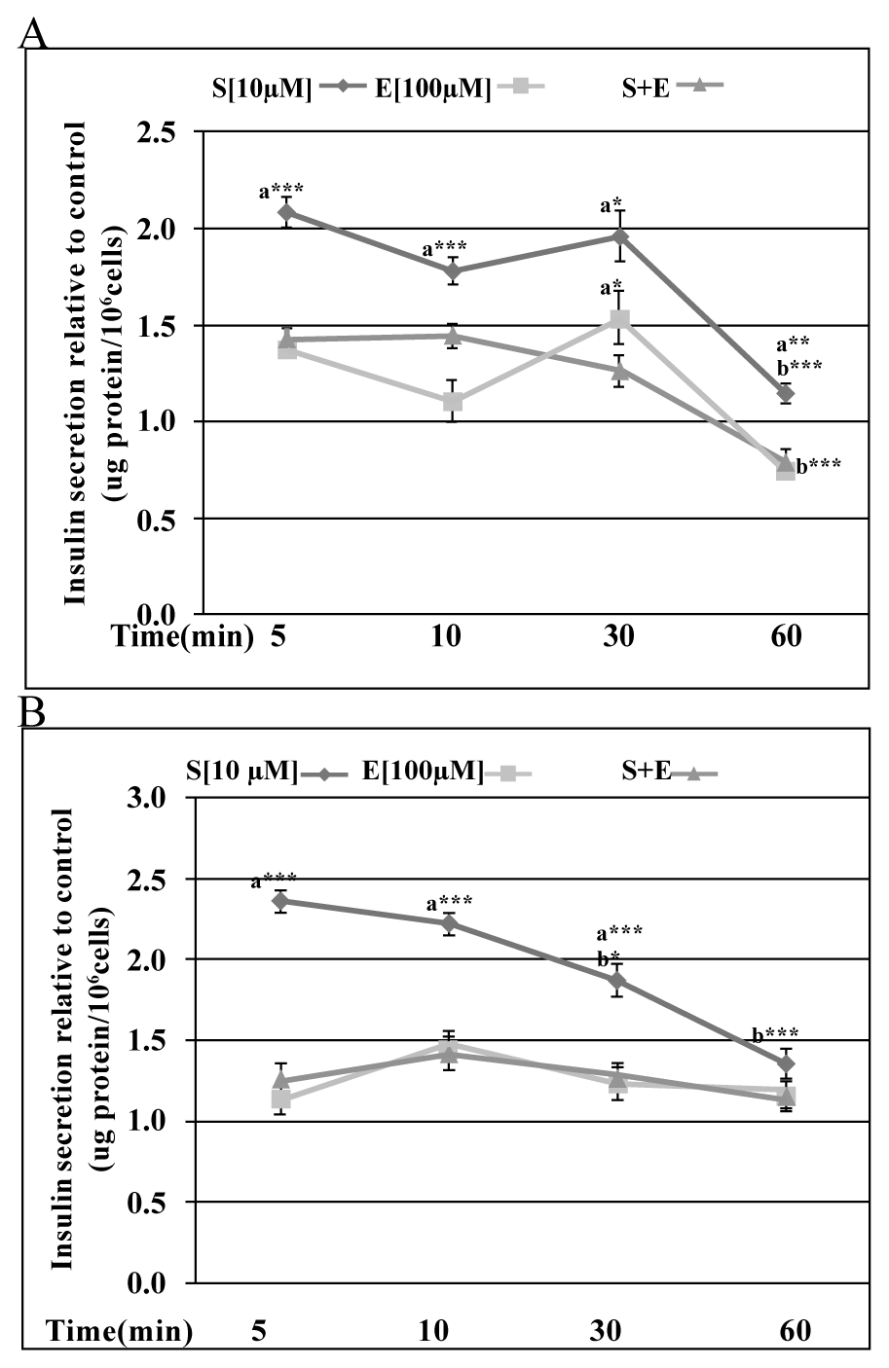
Effects of Efaroxan (E) on S43126(S)-induced insulin release. I_1_-imidazoline receptor antagonist Efaroxan) [100 μM] attenuated the insulinotropic response of Min6 cells to S41326 under (2A) low and (2B) high glucose. (2A) Data are presented as mean percent change +/− standard error from three separate experiments run in duplicate. (***a***= **p< 0.01, ***p< 0.001 same time point, compared to **E** and ***b***= ***p< 0.001 same dose, different time compared to 5 mins, ANOVA). (2B) Data are presented as mean percent change +/− standard error from three separate experiments run in duplicate. (***a***= ***p< 0.001 same time point, compared to **E** and ***b***= ***p< 0.001 same dose, different time compared to 5 mins, two-way ANOVA).

In order to evaluate the role of I_1_-imidazoline receptor and L-type calcium channel in the insulinotropic effect of S43126, we treated Min 6 cells with S43126 [10^−5^M] in the presence or absence of either efaroxan [100 μM] (I_1_-antagonist) or nifedipine [10 μM] (L-type calcium channel blocker) for various times (5–60mins). We used S43126 [10^−5^M] as this dose produced the maximum release of insulin in our previous study. Under conditions of basal glucose, efaroxan caused 92%, 50%, 166%, and 85% inhibition of insulin release by S43126 at 5 mins, 10 mins, 30 mins, and 60 mins respectively (Figure 3A). Efaroxan by itself caused release of insulin under both basal and high glucose conditions. Under conditions of high glucose, efaroxan caused a greater than 89%, 100%, 93%, 100% inhibition of insulin release by S43126 at 5 mins, 10 mins, 30 mins and 60 mins respectively (Figure 3B).

**Figure 4:**
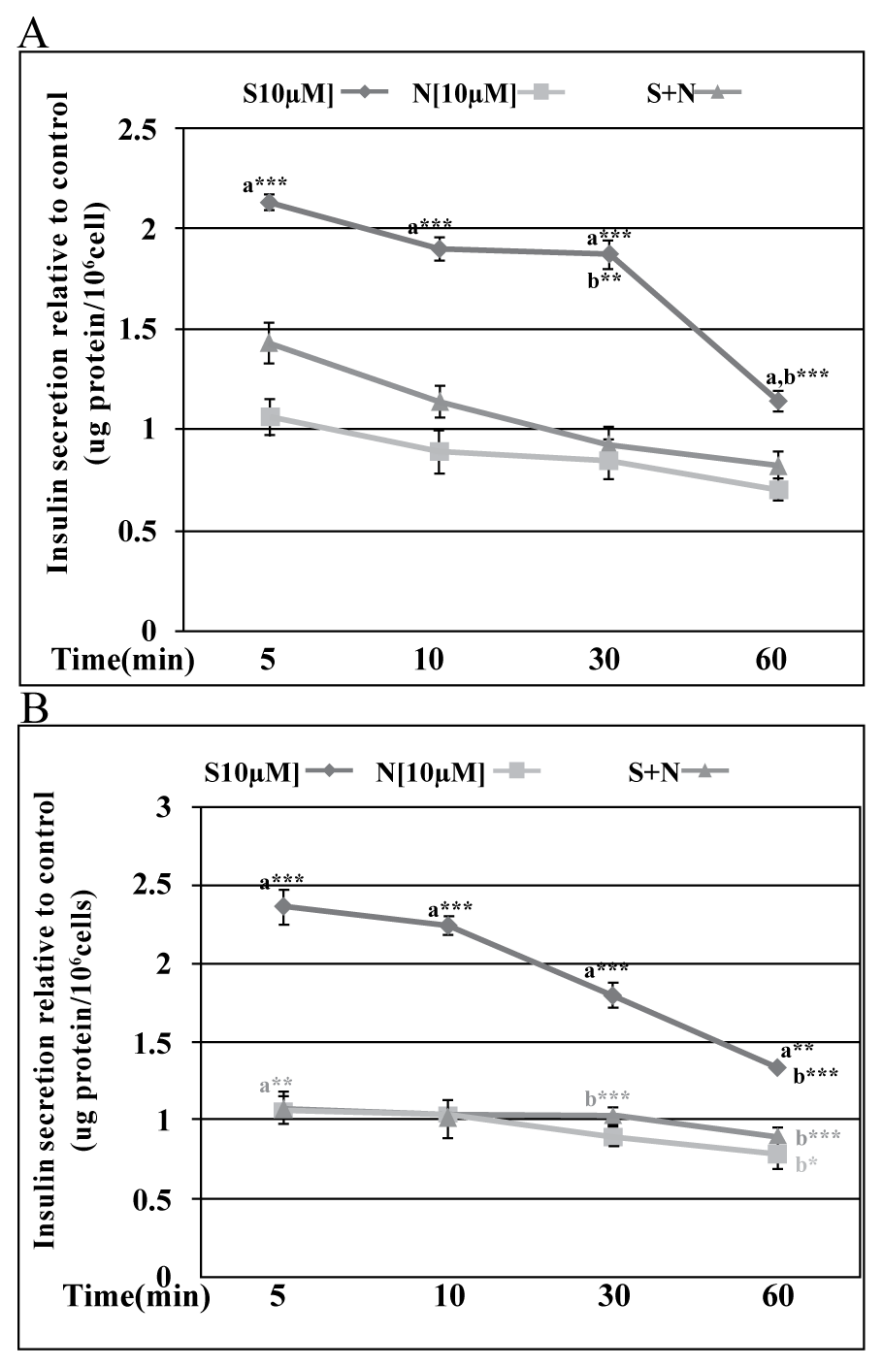
Effects of Nifedipine (**N**) on S43126(S)-induced insulin release. Voltage-gated L-type calcium channel blocker nifedipine [10 μM] attenuated the insulinotropic response of Min6 cells to S43126 under (3A) low and (3B) high glucose. (3A) Data are presented as mean percent change +/− standard error from three separate experiments run in duplicate. (***a***=*p< 0.05, ***p< 0.001 same time point, compared to **N** and ***b***= **p< 0.01, ***p< 0.001 same dose, different time compared to 5 mins, ANOVA). **(3B)** Data are presented as mean percent change +/− standard error from three separate experiments run in duplicate. (***a***= ***p< 0.001 same time point, compared to **N** and ***b***= **p< 0.01, ***p< 0.001 same dose, different time compared to 5 mins, two-way ANOVA).

Co-treatment of Min 6 cells with nifedipine [10μM], caused an approximately 73%, 63%, 91% and >80% reduction in insulin release at 5mins, 10 mins, 30 mins and 60 mins respectively under basal glucose conditions (Figure 4A). Inhibition of insulin release by nifedipine was more dramatic under high glucose conditions. Nifedipine caused a greater than 90% reduction in insulin release at all time points (Figure 4B).

### Effects of S43126 on [Ca^2+^]_i_

**Figure 5:**
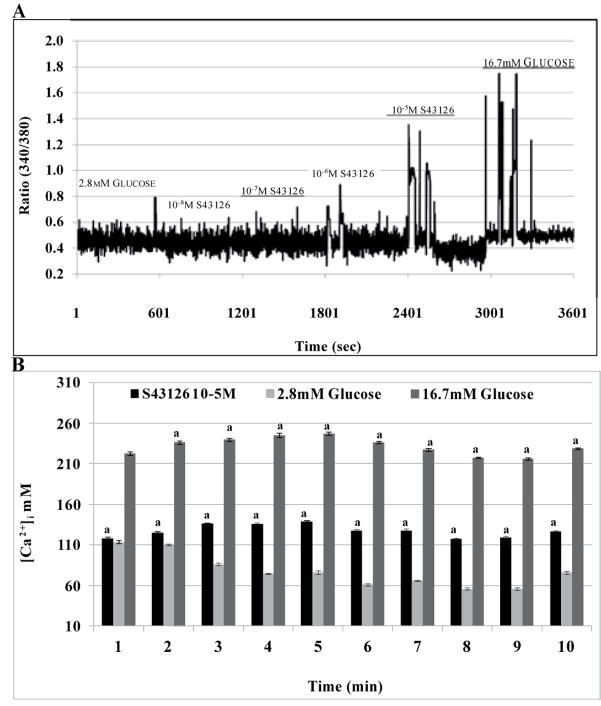
Effects of S43126 on Ca^2+^ oscillations. S43126 evoked a dose-dependent increase in cytosolic Ca^2+^ under (3A) conditions of basal glucose (2.8 mM). (B) S43126 [10^−5^M] caused an increase in [Ca^2+^]_i_ that was greater than the influx caused by 2.8 mM glucose but less than the influx caused by 16.7 mM glucose at all time points studied. Data are presented as mean percent change +/− standard error from eight separate experiments (***p< 0.001 2.8 mM Glucose (control) vs S43126 [10μM] & 16.7mM Glucose at the same time, one-way ANOVA).

When Min6 β-cells were incubated with S43126 [10^−8^M – 10^−5^M] in the presence of 2.8mM glucose, S43126 caused a dose-dependent increase in [Ca^2+^]_i_ which was first observed at [10^−6^M] of S43126 (Figure 5A). The amplitude of the calcium oscillations evoked by S43126 at 2.8mM glucose were less than those produced by 16.7mM glucose, but greater than control, at higher doses. The increase in [Ca^2+^]_i_ caused by S43126 [10^−5^M] and 2.8mM glucose were similar at 1 min, however S43126 [10^−5^M] caused a gradual increase in [Ca^2+^]_i_ up to 5min followed by a slight decline. Under conditions of basal glucose, [Ca^2+^]_i_ gradually declined with time. The temporal response of [Ca^2+^]_i_ to high glucose, mimic that of S43126 [10^−5^M] but with higher levels of [Ca^2+^]_i_. (Figure 5B).

### Effects of S43126 on ^45^Ca^2+^ uptake

**Figure 6:**
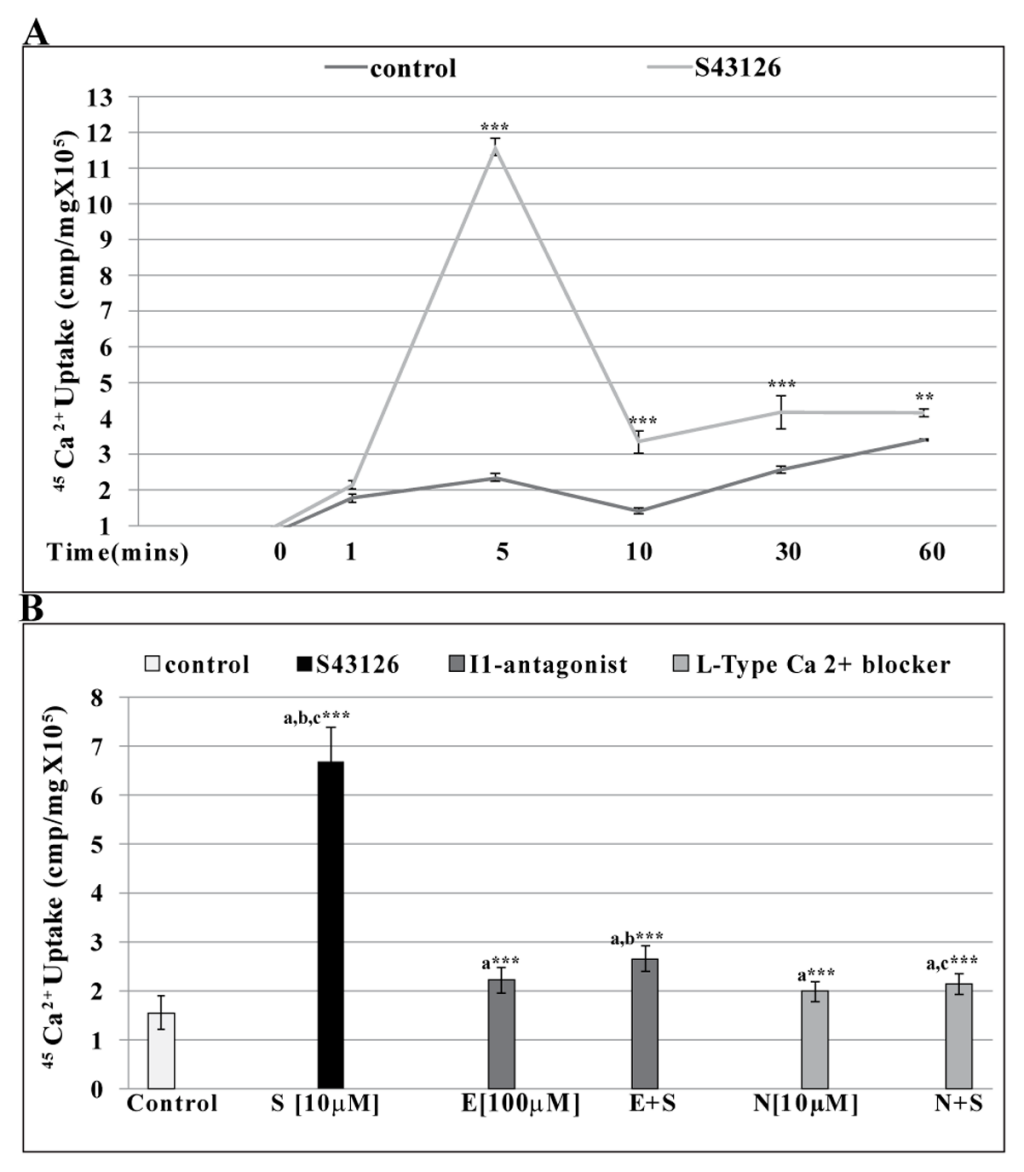
Effects of S43126 on ^45^Ca^2+^ uptake. (A) S43126 [10^−5^M] induced a time–dependent increase in ^45^Ca^2+^ influx up to 5mins, followed by a decline at 10 min and then a plateau between 30–60 mins. Data are presented as mean percent change +/− standard error from four separate experiments run in duplicate. (***p< 0.001 Control vs S43126 [10 μM] at the same time, two-way ANOVA). (B) The ^45^Ca^2+^ influx mediated by S43126 [10^−5^M] was attenuated in the presence of efaroxan [100 μM] and nifedipine [10 μM]. The data are presented as mean percent change +/− S.E. from four separate experiments run in duplicate. (***a***= ***p< 0.001 Control vs **S, E**, **E+S, N, N+S**, ***b***= ***p< 0.001 **E vs S, E+S** and ***c***= ***p< 0.001 **N vs S, N+S,** one-way ANOVA).

Min 6 cells were also incubated with ^45^Ca^2+^ alone or in combination with S43126 [10^−5^M] at various times (0–60 mins). There was a time–dependent increase in ^45^Ca^2+^ influx up to 5 mins, followed by a decline at 10 mins and then a plateau between 30–60 mins (Figure 6A). The maximum influx of greater than 3 fold was seen at 5 mins.

Since S43126 [10^−5^M] provoked marked ^45^Ca^2+^ influx at 5 mins, we co-treated cells with S43126 [10^−5^M] in the presence or absence of either efaroxan (10 μM) or nifedipine (10 μM) with the aim of determining whether I_1_-imidazoline receptor or L-type calcium channels mediated the observed ^45^Ca^2+^ influx into Min 6 cells. S43126 increased ^45^Ca^2+^ influx by 4 fold and this increase was blocked by efaroxan (Figure 6B). In addition, nifedipine, a blocker of L-type calcium channels, reduced ^45^Ca^2+^ influx by S43126 to near basal levels (Figure 6B).

### Effects of S43126 on ERK1/2, PKB phosphorylation and IRAS protein expression

**Figure 7:**
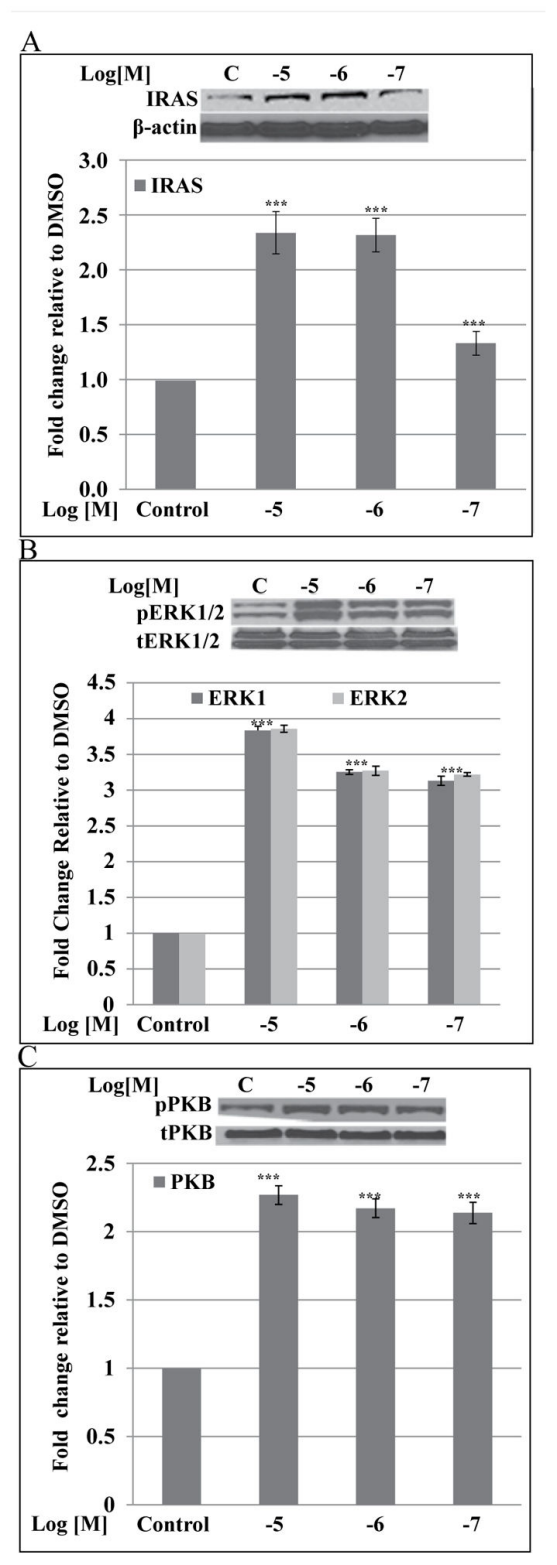
Dose-Response WB. Effects of S43126 [10^−7^ 10^−5^M] on (A) IRAS protein expression (B) ERK1/2 phosphorylation (C) PKB (Akt) phosphorylation. S43126 [10^−7^ 10^−5^M] caused an increased induction of IRAS protein, and a dose-dependent increase in the phosphorylation of both ERK1/2 and PKB. The relative protein expression of IRAS was defined by the ratio of the β-actin while the relative phosphorylation of ERK1/2 and PKB was defined by the ratio of phosphorylated active form to total immunoreactive protein, in arbitrary absorbance units. Data are presented as mean percent change +/− standard error from two separate experiments run in duplicate. (***p< 0.001 Control vs Dose [10^−7^–10^−5^M], one-way ANOVA).

S43126 [10^−7^M–10^−5^M] induce IRAS protein expression in a dose-dependent manner (Figure 7A). S43126 [10^−7^M–10^−5^M] also cause a 3-fold increase in ERK1/2 phosphorylation (Figure 7B) and a 2-fold increase in PKB phosphorylation (Figure 7C). ERK1/2 and PKB are components of both the insulin and imidazoline receptor signaling pathways.

## Discussion

Compounds that have the ability to treat both hypertension and insulin resistance would greatly improve compliance among patients. The novel imidazoline compound S43126 has been shown previously to lower blood pressure in rats following injection into the RVLM, but did not contract rat tail artery [23]. We therefore wanted to determine whether S43126 also had the ability to affect glucose homeostasis. Treatment of Min6 β-cells with S43126 caused a dose-dependent increase in insulin secretion at 5 mins, 10 mins and 30 mins under conditions of basal glucose (Figure 2A), and under high glucose (Figure 2B). This increase in insulin secretion was modest, but glucose-dependent, with S43126 causing a greater insulin release at higher concentrations of glucose at 5 mins and 10 mins. This is in contrast to sulfonylurea drugs which demonstrate a strong insulinotropic effect at basal glucose concentrations. Agents that stimulate robust insulin secretion at basal levels of glucose are more likely to cause hypoglycemia in patients [27]. A more desirable therapeutic outcome would be achieved by agents such as S43126 that augment glucose-induced insulin secretion.

The effect of imidazoline compounds on glucose homeostasis is mediated by all three subtypes of imidazoline receptors. I_1_-imidazoline and I_3_-imidazoline agonists mediate insulin release, while I_2_-imidazoline agonists mediate insulin sensitization. It was shown previously that cerebral injections of agmatine caused a decrease in plasma glucose in STZ-diabetic rats, but plasma levels of glucose and insulin were not affected in normal rats [8]. This suggested that agmatine was acting through a non-insulin dependent mechanism, and did not cause insulin release in normal rats. The effects of agmatine on glucose levels were attenuated in a dose-dependent manner with the I_2_-imidazoline receptor blocker BU-224. It was subsequently shown that peripheral injections of agmatine into STZ-diabetic rats also lowered plasma glucose in a dose-dependent manner [9]. The proposed mechanism for the peripheral glucose lowering effects of agmatine involved secretion of β-endorphins from the adrenal medulla, secondary to activation of adrenal I_2_-imidazoline receptors. β-endorphins are known to decrease the gene expression of phosphoenolpyruvate carboxykinase (PEPCK), a key enzyme in gluconeogenesis [9]. Increased *GLUT4* gene expression was also involved in the glucose lowering effects of agmatine [9].

The novel compound S4321 has affinity at I_2_-imidazoline receptors, however it is unknown whether this agent acts as an agonist or antagonist at the I_2_-imidazoline receptors [22]. It is unlikely that the insulin release caused by S43126 was due to an action at I_2_-imidazoline receptors, since as stated above, agmatine which also has effects at I_2_-imidazoline receptors does not cause insulin release in normal rats. In addition, I_2_-ligands such as cirazoline and idazoxan were shown to cause insulin release from RIN-5AH insulinoma cell line, but not by their actions at I_2_-imidazoline receptors, since irreversible blockade of the I_2_-receptor by clorgyline did not attenuate the effects of these compounds on insulin secretion. The proposed mechanism for insulin release by these ligands was an action on K_ATP_ channels [28].

Imidazoline compounds are not uniform in the mechanism by which they stimulate insulin release from pancreatic beta cells. Some imidazolines such as efaroxan [29,30], phentolamine [31] and RX871024 [32–33] stimulate insulin secretion both *in vivo* and *in vitro* by binding to K_ATP_ channels [29–34]. The pore forming subunit of the K_ATP_ channel, Kir6.2 contains an imidazoline binding site [35] to which certain imidazolines bind, leading to membrane depolarization, activation of voltage-dependent calcium channels, and subsequent calcium influx, and insulin secretion. Some imidazolines such as BL11282 stimulate insulin secretion only at elevated levels of glucose and does not block the K_ATP_ channel [18]. This property suggests that the risk of hypoglycemia would be very low in patients treated with BL11282. However, we are unaware of any published data suggesting that BL11282 lowers blood pressure. Thus BL11282 may not possess the utility that S43126 has in impacting both hypertension and insulin resistance.

S-21663 is an imidazoline compound that is a non glucose-dependent insulin secretagogue, which does not readily induce hypoglycemia [15]. The authors explained the discrepancy between the ability of S-21663 to stimulate insulin secretion at all levels of glucose and the lack of hypoglycemia seen *in vivo* by the existence of some compensatory mode of action by this compound *in vivo,* but not seen *in vitro*. S-21663 acts in a manner that is dependent on calcium entry via L-type calcium channels that are activated by closure of non K_ATP_ potassium channels [15]. Our novel compound S43126 also caused an increase in intracellular calcium via influx through nifedipine sensitive calcium channels. Unlike other imidazolines, we showed that the insulinotropic effect of S43126 could be blocked by efaroxan, an I_1_-imidazoline antagonist. In addition to its antagonistic effect at I_1_-imidazoline sites, efaroxan can also interact with K_ATP_ channels or the I_3_-imidazoline receptor to cause insulin release. We observed that efaroxan did cause release of insulin presumably through its action at K_ATP_ channels or the I_3_-imidazoline receptor. Combination of S43126 and efaroxan resulted in inhibition of insulin release; this suggested that at 100 μM, efaroxan acted as an effective blocker of the I_1_-receptor. The degree of antagonism caused by efaroxan was complicated by its ability to release insulin and a purer antagonist would prove more useful. The functional effects of S43126 at the various subytpes of imidazoline receptors need to be further studied, using inhibitors without intrinsic effects at these receptors.

S43126 also caused an increased induction of IRAS protein, and an increased phosphorylation of ERK and PKB which are components of the I_1_-imidazoline signaling pathways. In one study, PKB function in beta cells was disrupted by expression of a kinase-dead dominant negative form of PKBα (rip-*kdpkb*) and defective insulin secretion was observed, but no reduction in islet size [36]. This suggests that at least PKBα may play a role in insulin secretion. There is some controversy surrounding the exact role of different isoforms of PKB in the pancreas. Recent work by Buzzi et al. [37] showed that only PKBα but not PKBγ or PKBβ is activated downstream of IRS2 in beta cells. Overexpression of PKBα by adenovirus caused an increased proliferation of beta cells, while overexpression of PKBγ and PKBβ were ineffective. In another study, it was shown that PKBα-deficient mice can show enhanced glucose tolerance in addition to improved beta cell function and higher insulin sensitivity in adipocytes. Further studies will be needed to determine the importance of increased phosphorylation of PKB by S43126 on insulin sensitivity, and any possible involvement of I_2_-imidazoline receptors. These studies will need to consider the specific isoforms of PKB, which, as the recent studies showed, may even have opposing effects [37].

In conclusion, S43126 is a novel compound that activated I_1_-imidazoline receptors but not α-adrenoceptors [23,38]. We have shown that S43126 induced insulin secretion in a dose and time dependent manner. Because S43126 increased insulin release at higher glucose levels compared to basal condition, the risk of hypoglycemia associated with the use of S43126 is diminished [9]. Thus, the present study showed that S43126 activated at least two distinct mechanisms to promote insulin release. One of these may involve binding to imidazoline I_1_-receptors, while a second arises from calcium influx due to L-type Ca^2+^ channels. More importantly, S41326 activated components of the I_1_-imidazoline signaling pathways, namely IRAS, ERK and PKB in Min6 β-cells have the potential to act as an insulin sensitizer. Imidazoline agonists should be developed as an innovative pharmacological approach for the management of Type II diabetes.
